# Molecular characterization and immune role of TLR7 in *Labeo rohita*

**DOI:** 10.3389/fimmu.2025.1555048

**Published:** 2025-04-25

**Authors:** Saswati Pani, Bristy Ganguly, Smruti Mahapatra, Smruti Prajnya Dash, Rakesh Das, Ashis Saha, Mrinal Samanta

**Affiliations:** ^1^ Immunology Laboratory, Fish Health Management Division (FHMD), Indian Council of Agricultural Research - Central Institute of Freshwater Aquaculture (ICAR-CIFA), Bhubaneswar, Odisha, India; ^2^ Reproductive Physiology and Endocrinology Laboratory, Fish Nutrition and Physiology Division (FNPD), Indian Council of Agricultural Research - Central Institute of Freshwater Aquaculture (ICAR-CIFA), Bhubaneswar, Odisha, India

**Keywords:** TLRs, TLR7, Type-I IFN, PAMPS, *Labeo rohita*, RBCs, PBLs, innate immunity

## Abstract

**Background:**

Toll-like receptors (TLRs) play a vital role in the immune response by recognizing pathogen-associated molecular patterns (PAMPs) and triggering signaling pathways that activate innate immunity. In bony fish, TLR7 is essential for both antiviral and antibacterial defense; however, its interactions with a wide range of ligands and pathogens are still not well understood across various fish species. This study focuses on the identification and characterization of TLR7 in *Labeo rohita* (LrTLR7) and aims to evaluate its response to pathogen challenges and stimulation by PAMPs.

**Methods:**

To clone the TLR7 gene, RNA was extracted from *L. rohita* kidney tissue using a standard protocol, followed by cDNA synthesis with commercial kits. The TLR7 gene was amplified by PCR, and the gel-purified product was cloned into the pGEM-T Easy vector. DNA sequencing and BLAST analysis confirmed the identity of the LrTLR7 gene. The ORF of LrTLR7 cDNA was predicted using ORF-finder, while structural motifs in the encoded protein were identified through SMART. Phylogenetic relationships were analyzed using MEGA7 to construct evolutionary trees. Gene expression profiles of LrTLR7 were evaluated by quantitative real-time PCR (qRT-PCR) across developmental stages, tissues/organs of rohu fingerlings, and during challenges with *A, hydrophila* and *E. tarda* infections, as well as LPS and Poly I:C stimulation. Mucosal RBCs and PBLs were isolated using density-gradient centrifugation with HiSep™ LSM 1077 (Himedia, India). Cultured *L. rohita* gill (LRG) cells in Leibovitz’s L-15 medium were infected with *A. hydrophila* or *E. tarda* at a multiplicity of infection (MOI) of 1, following established protocols.

**Results:**

LrTLR7 showed the closest phylogenetic affinity to TLR7 in *Cyprinus carpio*. During embryonic development, LrTLR7 expression surged dramatically (~111-fold, *p*<0.05) in embryos at 120 h post-fertilization (hpf). In *L. rohita* juveniles, the gene was ubiquitously expressed across tissues/organs, with peak expression in gills (~2,000-fold). Following infection with *A. hydrophila* or *E tarda*, LrTLR7 gene transcripts in the liver increased sharply at 6 hpi (~93-fold and ~53,000-fold, respectively). In the infected fish, mucosal RBCs showed a ~500,000-fold upregulation (*p*<0.05), while PBLs exhibited maximal responses at 24 hpi (~5,000-fold for *A. hydrophila* and ~10 million-fold for *E. tarda*). In the LRG cell line, LrTLR7 gene expression rose ~30-fold by 3 hpi. during *A. hydrophila* infection. *In-vivo* stimulation with LPS or poly I:C triggered a ~30,000-fold increase in hepatic LrTLR7 expression at 12 h post-stimulation, with kidney tissue showing secondary activation. Mucosal RBCs and PBLs displayed rapid (1–3 h) LrTLR7 upregulation following *in-vitro* ligand exposure. Imiquimod and gardiquimod activated LrTLR7-signalling pathways in both *in-vivo* and *in-vitro* systems, elevating transcription of IRF7 and type I interferon genes.

**Conclusion:**

Similar to higher vertebrates, LrTLR7 plays a crucial role in responding to pathogenic invasions and various PAMPs to induce innate immunity. Consequently, TLR7 in fish represents a significant target for immune activation using specific agonists or ligands, which could aid in the prevention of fish diseases.

## Highlights

- Structurally, LrTLR7 consists of a signal peptide, 16 LRRs, and a TIR domain.
- LrTLR7 gene is expressed during embryogenesis and in various organs of rohu.
- LrTLR7 responds against *A. hydrophila* and *E. tarda* infection and PAMPs stimulation.
- RBCs and PBLs express LrTLR7 following LPS and poly I: C stimulation.
- Imiquimod and gardiquimod stimulation activate the LrTLR7-IRF7-Type-I IFN-signaling pathway.

## 1 Introduction

Innate immunity serves as the first stage of defense in the host and plays a crucial role in providing resistance against a broad spectrum of invading pathogens. It is therefore referred to as the primary defense mechanism of the hosts (1). Like other animals, fish also protect themselves against various microbial infections mostly with the help of innate or non-specific immunity (2). This broadly acting and quick defense of the host is primarily facilitated by pattern recognition receptors (PRRs), which are germ-line encoded and include the toll-like receptors (TLRs), nucleotide-binding and oligomerization domain (NOD)-like receptors (NLRs), and retinoic acid-inducible gene I (RIG-I)-like receptors (RLRs). These receptors play a crucial role in sensing various pathogens or pathogen-associated molecular patterns (PAMPs), such as flagellins, lipopolysaccharides (LPS), peptidoglycan (PGN), lipoproteins, lipoteichoic acid (LTA), and nucleic acids (DNA/RNA) (1, 3).

Structurally, TLRs are Type-I transmembrane glycoproteins characterized by extracellular leucine-rich repeat (LRR) domains, a transmembrane (TM), and a cytoplasmic Toll/IL-1 receptor (TIR) domain (4). On recognition of its ligand, the TIR recruits subsequent adaptor molecules that bind to downstream protein kinases, resulting in the production of Type-I interferons and proinflammatory cytokines that restrict further progression of the infection (5).

To date, 21 distinct TLRs (TLR1, TLR2, TLR3, TLR4, TLR5, TLR5S, TLR7, TLR8, TLR9, TLR13, TLR14, TLR18, TLR19, TLR20, TLR21, TLR22, TLR23, TLR25, TLR26, TLR27, and TLR28) have been reported in diverse fish species and are categorized into six families: (i) TLR1, (ii) TLR3, (iii) TLR4, (iv) TLR5, (v) TLR7, and (vi) TLR11 (6, 7). The TLR7 family mainly constitutes intracellular TLRs, which primarily recognize microbial nucleic acids as their ligands. They are further categorized into three crucial members, *viz.*, TLR7, TLR8, and TLR9. Both TLR7 and TLR8 bind to viral single-stranded (ss) RNA, whereas TLR9 is engaged in identifying the unmethylated CpG DNA present in the viral or bacterial DNA (8, 9). Like other vertebrates, TLR7, TLR8, and TLR9 have also been identified in various fish species such as zebrafish (*Danio rerio*) (10), pufferfish (*Takifugu rubripes*) (11), Atlantic salmon (*Salmo salar*) (12), rainbow trout (*Oncorhynchus mykiss*) (13), common carp (*Cyprinus carpio*) (14), catfish (*Ictalurus punctatus*) (15), large yellow croaker (*Pseudosciaena crocea*) (16), turbot (*Scophthalmus maximus*) (17), and Golden pompano (*Trachinotus ovatus*) (18).

TLR7 exhibits significant conservation throughout the vertebrate lineage, demonstrating the minimum rate of evolution in its LRR domains from primates to fish (19). In the higher vertebrates, TLR7 has been reported to recognize ssRNA, imidazoquinoline derivative R848, and guanine analogs such as loxoribine (8, 20). After recognizing its ligand, TLR7 moves to the endosome from the endoplasmic reticulum through the ER-resident membrane protein UNC93B1 (21, 22). Then, involving the myeloid differentiation primary response protein 88 (MyD88) as an adaptor molecule, it signals through nuclear factor (NF)-kappa B (kB) and interferon regulatory factor 7 (IRF7) pathways, resulting in the production of pro-inflammatory cytokines like interleukin (IL)-6 tumor necrosis factor (TNF)-α, and Type-I IFN (23–25). In the bony fish, TLR7 has been shown to play an essential role against viral and antibacterial immunity (26), but its response against a wide range of ligands and pathogens is mostly unknown in several fish species.

*Labeo rohita*, commonly referred to as “rohu,” is a vital species in freshwater aquaculture across South Asia, especially in India and Bangladesh. It is an economically significant fish species in global aquaculture, with an annual production exceeding 2 million tons. Its significance stems from its rapid growth, superior flesh quality, and strong consumer demand, making it integral to carp polyculture systems and a substantial contributor to the region’s fish production and economic landscape. To boost production levels, the farming of rohu is shifting from semi-intensive to intensive aquaculture methods, with production levels ~3 to 5 tons per hectare annually (27).

This transition to high-density farming increases the risk of encountering various fish pathogens, notably *Aeromonas* sp. (28–30), *Edwardsiella* sp. (31, 32), *Pseudomonas* sp., *Vibrio* sp. Argulus parasites (33), viral pathogens (34) etc., leading to infections, diseases, and significant fish mortality. To prevent these infections and diseases, broadly acting innate immunity facilitated by TLRs is anticipated to play a pivotal role. Hypothesizing the importance of TLRs in piscine innate immunity, this study on TLR7 in *L. rohita* (LrTLR7) was aimed for its identification, cloning, characterization and also to investigate its function during pathogen challenges and PAMPs stimulations.

In this article, we report the identification and molecular characterization of LrTLR7, along with its response to various PAMPs stimulations and infections with *A. hydrophila* and *E. tarda*. This article also elucidates the LrTLR7-signaling pathway by examining the expression of IRF7 and Type-I IFN response following imiquimod and gardiquimod stimulations. Collectively, these findings highlight the important immune role of LrTLR7.

## 2 Experimental methodologies

### 2.1 Ethical declaration

The use of live rohu fingerlings for the experiments was permitted by the Ethics Committee of the Indian Council of Agricultural Research (ICAR)-Central Institute of Freshwater Aquaculture (CIFA), Government of India (Approval number ICAR-CIFA/Eth/02/2016). The experimental protocols were also in line with the ARRIVE guidelines.

### 2.2 Fish

Healthy rohu fingerlings (avg. wt. ~70 g) were procured from the ICAR-CIFA and were stocked in 500-L fiber-reinforced plastic (FRP) tanks with continuous aeration. While conducting acclimatization of fish for 4 weeks, they were fed with commercial carp diet (CIFA-CARP GROWER, Agarwal Trading Corporation, India) twice a day along with water exchange two to three times a week. During this study, water temperature ranged between 26 and 28°C and pH ranged between 7.3 and 7.6.

For the collection of tissue samples, fishes were first anesthetized with 100 mg/L of anesthesia [1:1 w/w mixture of ethyl-3-aminobenzoate methane sulfonate (Sigma-Aldrich, Cat. No. E10521) and sodium bicarbonate (Sigma-Aldrich, Cat. No. S5761)], followed by dissection and sample collection.

### 2.3 Bacterial strains

*A. hydrophila* (ATCC-35654) and *E. tarda* (ATCC-15947) were separately cultured in the Luria broth (HiMedia, India) for 16 h at 37°C with continuous shaking. The cultures were centrifuged at 5,000 rpm for 10 min at 4°C, and the resulting pellet was then washed with phosphate-buffered saline (PBS, pH 7.2). The viable cell count of the bacterial cultures was assessed by counting the number of colony-forming units (CFU) following 10-fold serial dilutions and spreading on the Luria Bertani agar (HiMedia, India).

### 2.4 Cloning and characterization of LrTLR7

To clone the TLR7 gene in rohu, total RNA was extracted from the kidney tissue and cDNA was prepared using 1 µg of RNA. Then, several primers (**Table 1**) were designed from the predicted TLR7 gene sequences (GenBank Acc no.: XM_051118575.1). In a 50-µl reaction, 1 µl of the rohu-kidney cDNA was taken as the template, and for the PCR amplification, the following parameters were followed: initial denaturation at 95°C for 5 min, followed by 45 cycles of denaturation at 94°C for 30 s, annealing for 30 s (temperature was different for each set of primers), extension at 72°C for 1 min, and then a final extension at 72°C for 5 min. The resultant PCR products were analyzed in a 1% agarose gel. A single band with the desired size was purified from the gel with the agarose gel DNA extraction kit (Roche, Germany, Cat No. 15852400). Subsequently, the purified DNA was cloned with the pGEM-T Easy vector (Promega, USA). Recombinant plasmids were isolated and DNA-sequenced with T7 and SP6 primers at Barcode Biosciences Pvt. Ltd., Karnataka, India. Obtained DNA sequences were analyzed by BLAST search and were confirmed as partial sequences of the LrTLR7 gene. Several partial LrTLR7 cDNA sequences were aligned to obtain the full-length LrTLR7 cDNA sequence.

**Table 1:** Primers, their nucleotide sequence and their application in this study.

Primers	Nucleotide sequence (5`-3`)	Application
**TLR7 FW1**	CGAACGTCAAAAAGAATCCCAGA	Cloning
**TLR7 RV1**	GGCCTCGTTCAGTGCAGTCG	
**TLR7 FW2**	TGCCGGTCTCATCTCTTTTCTGG	
**TLR7 RV2**	CCGAAGGGATGTGGGTTAGAGAG	
**TLR7 FW3**	TGCCCAAATAATGCTCCACTTCA	
**TLR7 RV3**	GGCGTTCCCAGAGAGATTCAAA	
**TLR7 FW4**	TTCCCCAATGTCGCAAGGTG	
**TLR7 RV4**	AAAGGCCCTCCCAATGAAATGAC	
**TLR7 FW5**	CGACACGAGGTACGCCAGCTA	
**TLR7 RV5**	GCGGTATCCCTTGAGTTTGGC	
**TLR7 FW6**	CCCTGTCCATCATTCTCTGCATT	
**TLR7 RV6**	GGGTTCCTCGGCCACTCG	
**TLR7 FW7**	GCGATACATCAAGAGCGGCAG	
**TLR7 RV7**	TGCGGATAAAGTAAACAGGCTCG	
**TLR7 FW8**	TGGCCAAACTCAAGGGATACCG	Gene expression analysis by qRT-PCR
**TLR7 RV8**	GGTGGGCCAAATAAAACGCTGT	
**β-actin FW**	AGACCACCTTCAACTCCATCATG	
**β-actin RV**	TCCGATCCAGACAGAGTATTTACGC	
**IRF7 FW**	CTGAGAGGGGAGCAAATACG	
**IRF7 RV**	TGTCCTGACGAAAGCCATAGAT	
**Type- I IFN FW**	CGCTTGCAGATGGCTCGACAG	
**Type -I IFN RV**	TGGCCTCTTTTGGTATGGGTCCT	

### 2.5 Characterization of LrTLR7 cDNA and protein

The ORF of LrTLR7 cDNA was predicted through ORFfinder (http://www.ncbi.nlm.nih.gov/orffinder/), and the molecular weight was determined by Expasy tool (http://web.expasy.org/cgi-bin/compute_pi/pi_tool). To identify the structural motifs such as signal peptide (SP), LRR, and TIR domains in the TLR7, amino acid sequences of TLR7 of various fish species were retrieved from the NCBI GenBank database (http://www.ncbi.nlm.nih.gov/protein) and were analyzed with SMART (Simple Modular Architecture Research Tool) (http://smart.embl-heidelberg.de). To investigate the phylogenetic relationship among TLR7 proteins, multiple-sequence alignment data of TLR7 was generated through the Clustal Omega program (http://www.ebi.ac.uk/jdispatcher/msa/clustalo), and the phylogenetic tree was generated via the neighbor-joining method of the MEGA7 (Molecular Evolutionary Genetics Analysis) program.

### 2.6 Isolation and *in-vitro* culture of mucosal RBCs and PBLs

To obtain mucosal RBCs, gills were dissected from the rohu fingerlings and were kept in a petri dish with ~5 ml of PBS and 100 μl of 0.5 M EDTA, and then RBCs from the gill filaments were carefully scrapped out. For the purification of RBCs, 750 μl of HiSep™ LSM 1077 (HiMedia, India, LS001-100ML) was taken in a 1.5-ml Eppendorf tube, and on top of it, the same volume of the scrapped-out RBC suspension was layered carefully under aseptic condition. Then, centrifugation was performed at 400g (Eppendorf centrifuge, 5430R) for 30 min at 20°C. Following centrifugation, the uppermost layer was removed and the RBCs collected at the bottom were washed twice with PBS following centrifugation at 100g for 10 min at 20°C. Purified RBCs were cultured in RPMI 1640 medium (Sigma, Germany, Cat No. R8758) containing 10% fetal bovine serum (FBS) (HiMedia, India), 1 mM sodium pyruvate (Gibco, USA), and 1% antibiotic and antimycotic solution (Sigma, Germany, Cat No. A5955).

Similarly, PBLs were isolated from the blood of rohu fingerlings, following the density gradient centrifugation with HiSep™ LSM 1077 (35). At first, blood was collected from the caudal vein using a 2-ml sterile hypodermic syringe (Dispovan, India) that had been pre-rinsed with 0.5 M EDTA. The drawn blood was collected in a K2-EDTA-coated blood collection tube (RANKEM, India, Cat. No. 120419), and an equal volume of PBS was added to it and mixed gently. This suspension was layered over an equal volume of HiSep™ LSM 1077 gradient and centrifuged at 400g for 30 min at 20°C. Then, the interface and band obtained were collected in a new fresh tube, washed twice with PBS, and cultured in L(Leibovitz)-15 medium (Gibco, USA, Cat No. 11415-064) supplemented with 10% FBS and 1% antibiotic and antimycotic solution (Sigma, Germany, Cat No. A5955).

### 2.7 Cell staining and counting

Purified mucosal RBCs and PBLs were diluted in their respective media. A very small amount (~50 μl) of diluted cells was taken on a clean glass slide, and a thin and even smear was prepared and allowed to air-dry. A few drops of undiluted Giemsa stain (Merck, Germany) were poured on it, and after 2 min, a few drops of nuclease-free water (HiMedia, India) were added. After 3 min, the slide was rinsed with distilled water, air-dried, and examined under the oil immersion microscope (100×). The population of live and dead cells was determined by mixing equal volumes (10 μl) of diluted cells and trypan blue dye and then loading it on a disposable cell counting slide (Cat. No. 1450015; Bio-Rad, USA). The prepared slide was then analyzed through a TC20 automated cell counter (Bio-Rad, USA).

### 2.8 Ontogenic expression of LrTLR7

To investigate the expression profile of the LrTLR7 gene during embryonic development, induced breeding was conducted at the ICAR-CIFA carp hatchery. Following egg release and fertilization, samples were collected in TRI reagent (SIGMA, USA, Cat. No. T9424) at various stages of their development (0 h—fertilized egg stage, ~6 h—pharyngula stage, ~9 h—hatched spawns, and ~24, ~48, ~72, and ~120 h—advanced stages of spawn development) (36). The stages of embryonic development were confirmed by microscopic (10×) (Zeiss, Germany) examination.

### 2.9 Basal expression analysis of LrTLR7

To examine the basal expression of the LrTLR7 gene, healthy rohu fingerlings (N=3) were anesthetized, and various organs/tissues such as the eye, brain, muscle, skin, gill, intestine, heart, kidney, spleen, liver, and blood were collected in TRI reagent followed by RNA extraction, cDNA preparation with 1 µg of RNA, and LrTLR7 gene expression analysis by qRT-PCR (quantitative real-time PCR).

### 2.10 Bacterial infection

Rohu fingerlings were divided into two categories: (a) uninfected and (b) infected for 6, 12, 24, and 36 h with each category containing three fish (N=3). For infection, 100 µl PBS containing *A. hydrophila* or *E. tarda* (1×10^6^ CFU/ml) was intra-peritoneally (i.p.) injected into each fish, whereas the control group of fish received only PBS. After the designated period of infection, the fish were anaesthetized and various immunologically important tissues (gill, liver, kidney, and blood) along with mucosal RBCs and PBLs were collected separately in 1 ml TRI reagent. Thereafter, RNA was extracted and 1 µg of it was used for cDNA preparation and LrTLR7 gene expression by qRT-PCR.

### 2.11 Expression of LrTLR7 gene in response to PAMPs/ligands stimulation

#### 2.11.1 *In-vivo* stimulation

To study the *in-vivo* response of the LrTLR7 gene against various PAMPs, rohu fingerlings were categorized into the (a) unstimulated group and the (b) stimulated group with PAMPs (LPS and Poly I: C), each group consisting of three fish (N=3). The stimulated group of fishes was further sub-categorized into (i) 6 h, (ii) 12 h (iii) 24 h, and (iv) 36 h. For LPS stimulation, purified LPS of *Escherichia coli* (serotype O111: B4) (Cat No. L3024-5MG, SIGMA, Germany) was first mixed with the endotoxin-free water at a concentration of 5 μg/μl. Subsequently, each fingerling was injected with 100 µl of PBS containing 50 µg of LPS, whereas only 100 µl of PBS was administered to the fingerlings in the unstimulated group.

Similarly, for the poly I:C stimulation, poly I:C (Sigma, USA) was first reconstituted in the DEPC-treated water at a concentration of 10 mg/ml to make the stock solution. Then, 20 μl of this stock solution of poly I:C was added in 80 μl of the DEPC-treated water and mixed properly. Then, this 100 µl of the DEPC-treated water containing 200 µg of poly I:C was i.p. injected into each of the rohu fingerlings, whereas the unstimulated group of fish were injected with 100 µl of DEPC-treated water only.

To stimulate rohu fingerlings with the imiquimod (Sigma-Aldrich, Cat. No. I5159) and gardiquimod (Sigma-Aldrich, Cat. No. SML0877), these ligands were separately reconstituted in PBS, and then 100 µl of PBS containing 10 µg of either imiquimod or gardiquimod was i.p. injected to each of the fingerling (N=3). The unstimulated group of fish was i.p. injected with 100 µl of PBS only.

After 6, 12, 24, and 36 h, gill, liver, kidney, and blood were collected from both unstimulated and stimulated groups of fingerlings and were collected in TRI reagent for the extraction of RNA followed by cDNA synthesis using 1 µg of RNA and LrTLR7 gene expression analysis by qRT-PCR.

#### 2.11.2 *In-vitro* stimulation

To examine the response of the LrTLR7 gene in the mucosal RBCs and PBLs, these cells were purified from the rohu fingerlings and were cultured *in-vitro* followed by LPS or poly I:C stimulation. At first, purified cells were distributed in a 24-well cell culture plate (Cat. No. 142475; Nunc, Denmark) at a concentration of 10^7^ cells/ml/well and were categorized into (a) unstimulated control, (b) 1 h stimulated, and (c) 3 h stimulated groups. RBCs were stimulated with LPS at a concentration of 10 µg/ml/well or with poly I:C at 100 µg/ml/well. Similarly, for the PBL stimulations, the concentration of poly I:C remained the same as that of RBCs, but the concentration of LPS was changed to 20 µg/ml/well. Following stimulation with the PAMP/ligands, the plates containing cells were incubated at 28°C. After the designated time, cells were harvested, and RNA was extracted followed by cDNA synthesis with 1 µg of RNA and LrTLR7 gene expression analysis by qRT-PCR.

To study the modulation of LrTLR7 gene expression in the LRG cell line (NRFC023) (37), these cells were seeded in a 6-well cell culture plate (BD Biosciences, Cat. No. 353046) at a concentration of 10^7^ cells/ml/well and were categorized into (a) unstimulated (control) and (b) stimulated groups. The stimulated group was further sub-categorized into four groups; (i) 6 h, (ii) 12 h, (iii) 24 h, and (iv) 36 h. In the stimulated group, LRG cells were stimulated with imiquimod or gardiquimod at a concentration of 10 µg/ml/well and then were incubated at 28°C. After the designated time interval, both control and stimulated cells were harvested, RNA was extracted, cDNA was synthesized with 1 µg of RNA, and LrTLR7 gene expression was analyzed by the qRT-PCR assay.

### 2.12 Infection of LRG cells with *A. hydrophila* and *E. tarda*

To analyze the response of LrTLR7 gene expression during the progression of bacterial infections, at first LRG cells were seeded in a 6-well cell culture plate in L-15 growth medium at 28°C. After 24 h, the spent L-15 growth medium were removed and the cells were washed with PBS. Thereafter, fresh L-15 growth medium without antibiotics was added to the wells. The LRG cells were grouped into two groups: (a) uninfected group and (b) infected group. In the infected group, LRG cells were infected with either *A. hydrophila* or *E. tarda* with 1 MOI (multiplicity of infection), whereas the control group of cells contained only L-15 growth medium. After 30 min of incubation, the medium was replaced with fresh L-15 growth medium without antibiotics. The cells were then observed microscopically for upto 3 h post-infection. LRG cells in the *A. hydrophila*-infected group were harvested in TRI reagent at 1 and 2 h 30 min post-infection, and from *E. tarda*-infected cells at 1 and 3-h post-infection. Thereafter, RNA was isolated, cDNA was prepared with 1 µg of RNA, and LrTLR7 gene expression analysis was carried out by the qRT-PCR assay.

### 2.13 RNA isolation and cDNA synthesis

RNA was extracted from the control, infected, or stimulated tissues, RBCs, PBLs, and LRG cells following the instructions of TRI reagent (Sigma-Aldrich, USA, Cat. No. T9424). Both the quality and concentration of the RNA in the samples were analyzed by a NanoDrop 2000 Spectrophotometer (Thermo Fisher, USA). The genomic DNA contamination in the RNA was eliminated by treating 1 µg of the total cellular RNA with 1 unit of DNase I (Thermo Fisher Scientific, USA). Taking 1 µg of RNA, the cDNA was synthesized using a RevertAid™ First-Strand cDNA Synthesis Kit (Fermentas, Cat No. K1622) along with oligo-dT and random hexamer primers.

### 2.14 Quantitative real-time PCR

The qRT-PCR analysis of the LrTLR7, IRF7, Type-I IFN, and β-actin (housekeeping) genes was conducted using the LightCycler^®^ 480 II real-time PCR detection system (Roche, Germany). Each qRT-PCR reaction was performed in duplicate wells, with each well containing a 10-µl reaction mixture composed of 3.5 µl PCR-grade water, 5 µl (2×) SYBR Green I master mix (Roche, Germany), 0.25 µl forward primer, 0.25 µl reverse primer, and 1.0 µl cDNA. The cycling conditions included a pre-incubation step at 95°C for 10 min, followed by 50 cycles of denaturation at 94°C for 10 s, annealing at 60°C for 10 s, and extension at 72°C for 10 s. A negative control reaction without cDNA was included to check the specificity of the reaction. After each PCR run, melting curve analysis and gel loading of selected samples were performed to verify the specificity of the amplified products.

For the relative quantification of the target genes (LrTLR7, IRF7 and Type-I IFN), their expression levels were normalized with β-actin (the housekeeping gene), and fold change was calculated using the 2^−ΔΔCT^ method (38). The data obtained from the qRT-PCR analysis are graphically presented from one experiment among the three separate experiments (N=3) along with their ± standard error (S.E.). A significant difference (p<0.05) in the LrTLR7, IRF7, Type-I IFN gene expression between the control and infected/stimulated samples at each time point was evaluated by the Student’s t-test in Microsoft Excel 2019.

## 3 Results

### 3.1 Cloning, characterization, and phylogenetic relationship of LrTLR7

**Figure 1:**
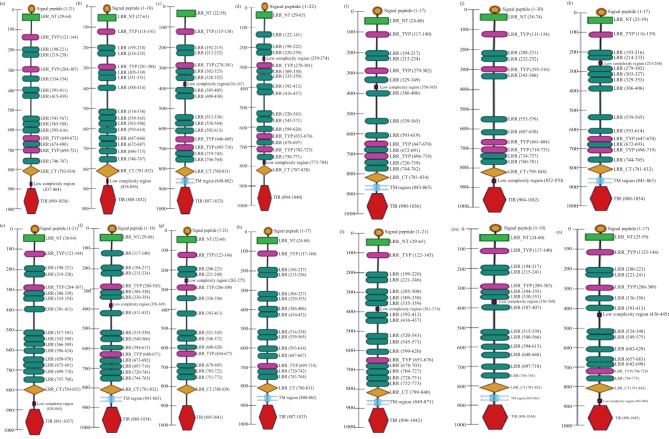
Schematic representation of various domains of TLR7 in different fish species. The SMART (Simple Modular Architecture Research Tool) program was used for predicting domain organization in TLR7 proteins of several fish species. The signal peptide (SP), leucine-rich repeat (LRR) domains, transmembrane domain (TM), and Toll/IL-1 receptor (TIR) domain are shown in their respective positions: **(a)** *Labeo rohita*; **(b)** *Danio rerio*; **(c)** *Siniperca chuatsi*; **(d)** *Mastacembelus armatus*; **(e)** *Cyprinus carpio*; **(f)** *Scophthalmus maximus L*.; **(g)** *Squaliobarbus curriculus*; **(h)** *Trachinotus blochii*; **(i)** *Oncorhynchus mykiss*; **(j)** *Salmo salar*; **(k)** *Takifugu rubripes*; **(l)** *Trematomus bernacchii*; **(m)** *Paralichthys olivaceus*; **(n)** *Ictalurus punctatus*.

**Figure 2:**
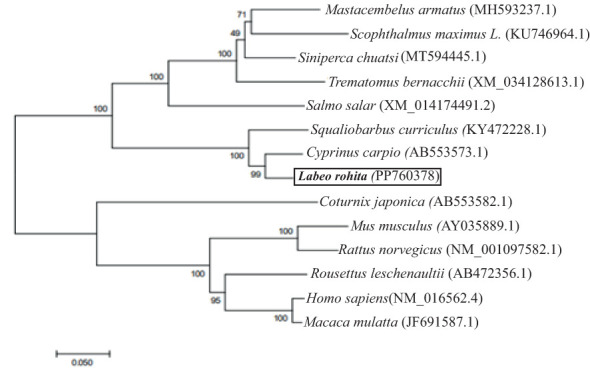
Phylogenetic relationship of LrTLR7 with TLR7 proteins of other animal species. Full-length amino acid sequences of TLR7 from various organisms were retrieved from the GenBank database, and aligned through the Clustal Omega. To generate a phylogenetic tree, the neighbor-joining method of the MEGA7 (Molecular Evolutionary Genetics Analysis 7) software was used. The evolutionary distances were computed using the Poisson correction method and were in the units of the number of amino acid substitutions per site. Branches in the tree were assessed for their reliability through bootstrap analysis with 1,000 replications, and depicted as percentage (%) on the branches.

The cloned LrTLR7 cDNA encoding the complete ORF has been submitted in the NCBI GenBank with the acc no: PP760378, and it consists of 3,147 nucleotides, translating into a polypeptide of 1,048 amino acids (aa) with a molecular mass of 120.708 kDa and pI of 8.13. Structurally, LrTLR7 protein comprises various significant domains such as SP (1–21 aa), LRR-NT (29–64 aa), 4 LRR-TYP (121–144, 284–307, 649–672, 698–721 aa) 10 LRR (198–221, 219–238, 334–354, 391–411, 415–439, 541–567, 565–588, 595–616, 674–690, 746–767aa), LRR-CT (783–834 aa), and a TIR domain (890–1036 aa). The structural relationship among various fish species TLR7 is shown in **Figure 1**. To establish the evolutionary relationship, a phylogenetic tree has been constructed with the TLR7 proteins from diverse species (**Table 2**), and the results revealed that within the fish species, LrTLR7 shared a close relationship with *C. carpio* and *S. curriculus* TLR7 proteins (**Figure 2**).

**Table 2:** TLR7 amino acids sequences of fish and other higher eukaryotes.

Organism	Scientific name	GenBank accession no
Fish	*Danio rerio*	XM_021479060.2
	*Siniperca chuatsi*	MT594445.1
	*Mastacembelus armatus*	MH593237.1
	*Cyprinus carpio*	AB553573.1
	*Scophthalmus maximus*	KU746964.1
	*Squaliobarbus curriculus*	KY472228.1
	*Trachinotus ovatus*	KU975046.1
	*Oncorhynchus mykiss*	GQ422119.1
	*Salmo salar*	XM_014174491.2
	*Takifugu rubripes*	AC156438.1
	*Trematomus bernacchii*	XM_034128613.1
	*Paralichthys olivaceus*	OQ594361.1
	*Ictalurus punctatus*	XM_017469254.3
Mammals	*Homo sapiens*	NM_016562.4
	*Mus musculus*	AY035889.1
	*Rousettus leschenaultii*	AB472356.1
	*Macaca mulatta*	JF691587.1
	*Rattus norvegicus*	NM_001097582.1
Bird	*Coturnix japonica*	AB553582.1
Amphibia	*Xenopus tropicalis*	NM_001127411.1

### 3.2 Ontogenic and basal expression profile of the LrTLR7 gene

**Figure 3:**
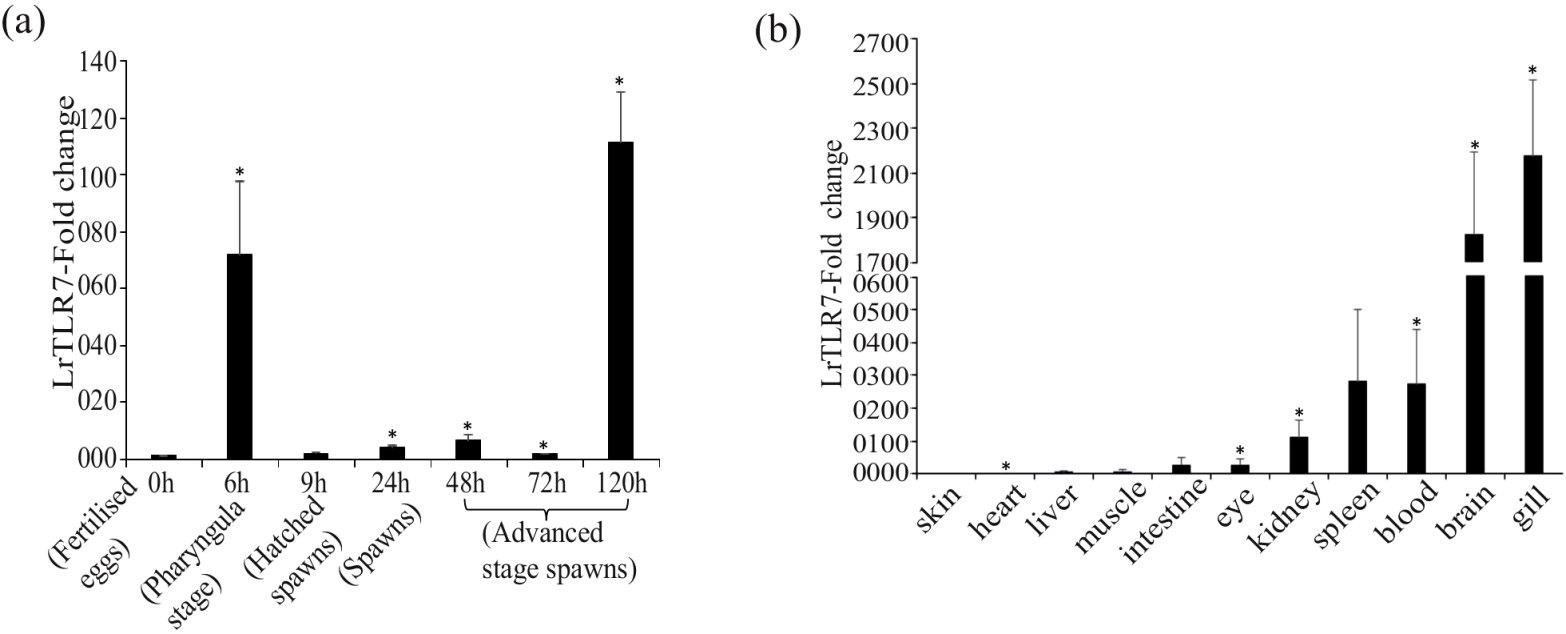
**(a)** LrTLR7 gene expressions in various embryonic developmental stages of *L. rohita.* Total RNA was extracted from different stages of embryonic development, and the expression of the LrTLR7 gene was analyzed by qRT-PCR. The expression of the LrTLR7 gene was represented as a ratio relative to β-actin (internal control) levels in the same samples. The gene expression at the fertilized egg stage (0 h) has been chosen as a calibrator (1), and the relative expression of LrTLR7 at various stages of development is represented as fold change from the calibrator. A representative data from three separate experiments (N=3) ± standard error (bars) has been presented. Significant difference (p<0.05) has been indicated with asterisks (*). **(b)** Basal expression of the LrTLR7 gene in various tissues of rohu fingerlings Total RNA was extracted from blood, brain, eye, gill, heart, intestine, kidney, liver, muscle, skin, and spleen, and qRT-PCR was carried out to investigate the expression of the LrTLR7 gene among the tissues. The expression of LrTLR7 gene transcript levels in each tissue has been represented as a ratio relative to β-actin (internal control) levels in the same samples. Among the tissues examined, skin expressed the lowest level of LrTLR7 and was chosen as the calibrator (1). The LrTLR7 gene expression in other tissues has been represented as fold changes from the calibrator. Representative data from three separate experiments (N=3) ± standard error (bars) have been presented. Significant (p<0.05) difference has been indicated with asterisks (*).

LrTLR7 gene expression was analyzed in various developmental stages, as well as tissues of rohu fingerlings by the qRT-PCR assay. The LrTLR7 gene was constitutively expressed across all the developmental stages, and the highest expression (~111-fold) was recorded at 120 h post-fertilization in the advanced stages spawns (**Figure 3a**). Among the tested organs/tissues, the basal expression of LrTLR7 gene was highest (~2,100-fold) in the gill tissues whereas the lowest expression was observed in the skin (**Figure 3b**).

### 3.3 *In-vivo* response of LrTLR7 gene against bacterial infection

**Figure 4:**
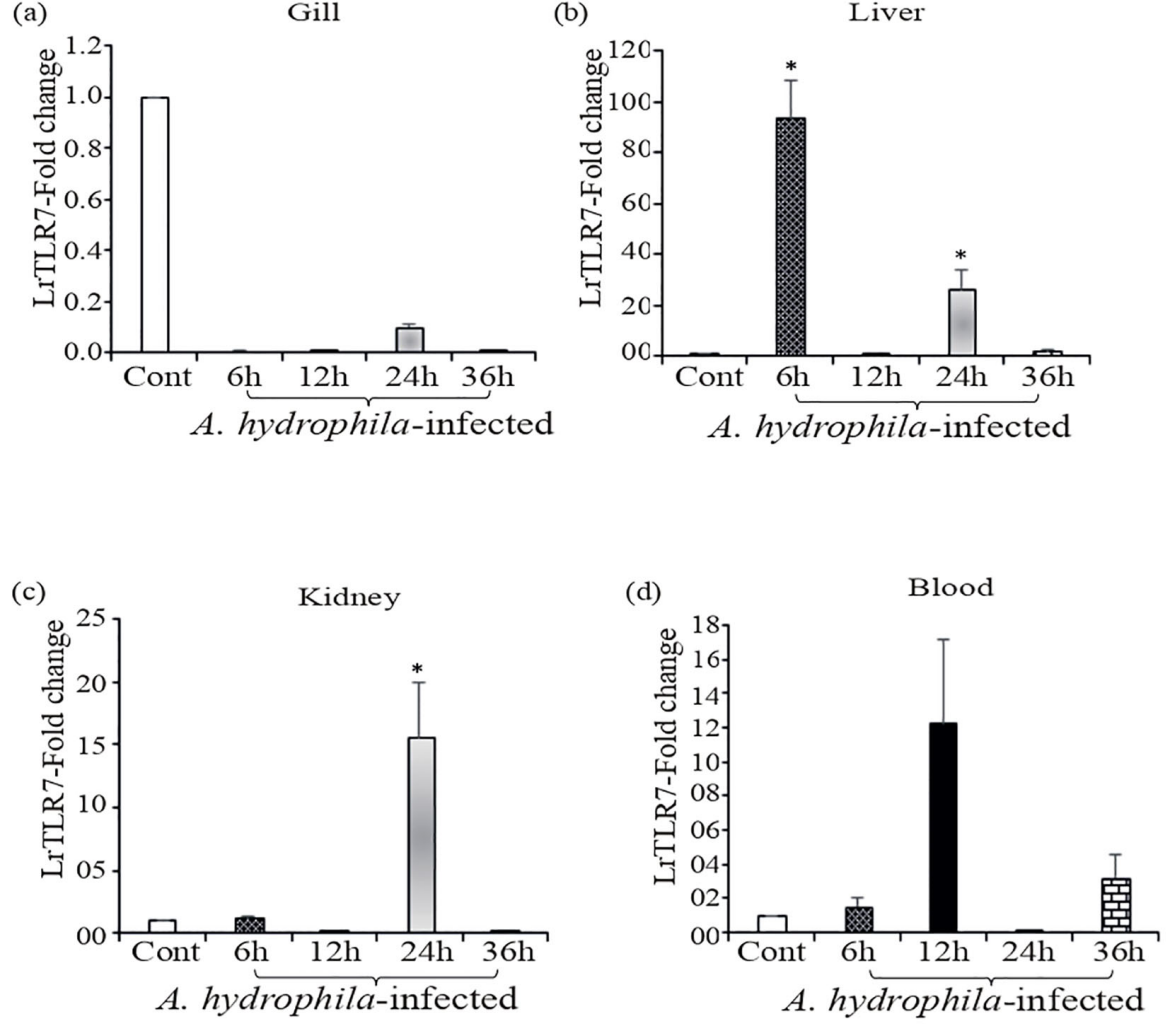
Expression of the LrTLR7 gene in response to *Aeromonas hydrophila* infection. Rohu fingerlings were either mock-infected (control) or infected with *A*. *hydrophila* (1×10^6^ CFU fish^−1^) by i.p. injection, and after the designated time course, total RNA was extracted from the control and infected fish tissues, cDNA was synthesized, and qRT-PCR was performed to analyze LrTLR7 gene expression. The expression of the LrTLR7 gene was analyzed, keeping β-actin as an internal control. Representative data of one experiment out of three separate experiments (N=3) are shown along with standard error (bars). Significant (p<0.05) differences in LrTLR7 gene expression between the control and infected fish groups are indicated with asterisks (*). LrTLR7 gene expression in *A*. *hydrophila*-infected fish **(a)** gill, **(b)** liver, **(c)** kidney, and **(d)** blood.

**Figure 5:**
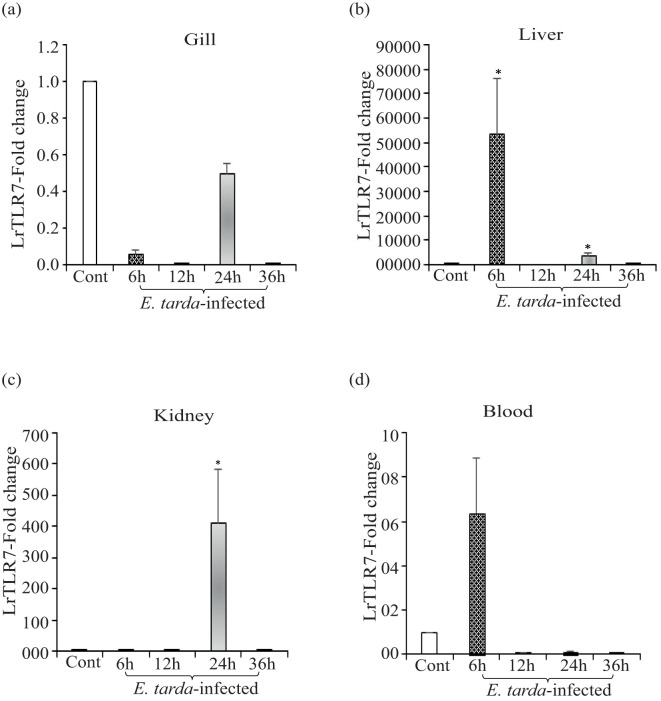
Expression of LrTLR7 gene in response to *Edwardsiella tarda* infection. Rohu fingerlings were either mock-infected (control) or infected with *E*. *tarda* (1×10^6^ CFU fish^−1^) by i.p. injection, and after the designated time course, total RNA was extracted from the control and infected fish tissues, cDNA was synthesized, and qRT-PCR was employed to analyze LrTLR7 gene expression. The expression of the LrTLR7 gene was analyzed keeping β-actin as an internal control. Representative data from one experiment out of three separate experiments (N=3) are shown along with standard error (bars). Significant (p<0.05) differences in LrTLR7 gene expression between the control and infected fish groups are indicated with asterisks (*). LrTLR7 gene expression in the *E*. *tarda*-infected fish **(a)** gill, **(b)** liver, **(c)** kidney, and **(d)** blood.

Compared with the uninfected control fish, in *A. hydrophila-*infected fish tissues, significant (p<0.05) upregulation of LrTLR7 gene expression at various time points was observed in the liver (6, ~93-fold, 24 h, ~26-fold), kidney (24 h, ~15-fold), and blood (6 h, ~2-fold, 12 h, ~12-fold, 36 h, ~3-fold), but in the gill, it was downregulated (**Figures 4a–d** **).** Similarly, in the *E*. *tarda* infection, LrTLR7 gene expression was also significantly (p<0.05) enhanced in the liver (6 h, ~53,602-fold), kidney (24 h, ~410-fold), and blood (6 h, ~6-fold) and remained downregulated in the gill tissues (**Figures 5a–d**).

**Figure 6:**
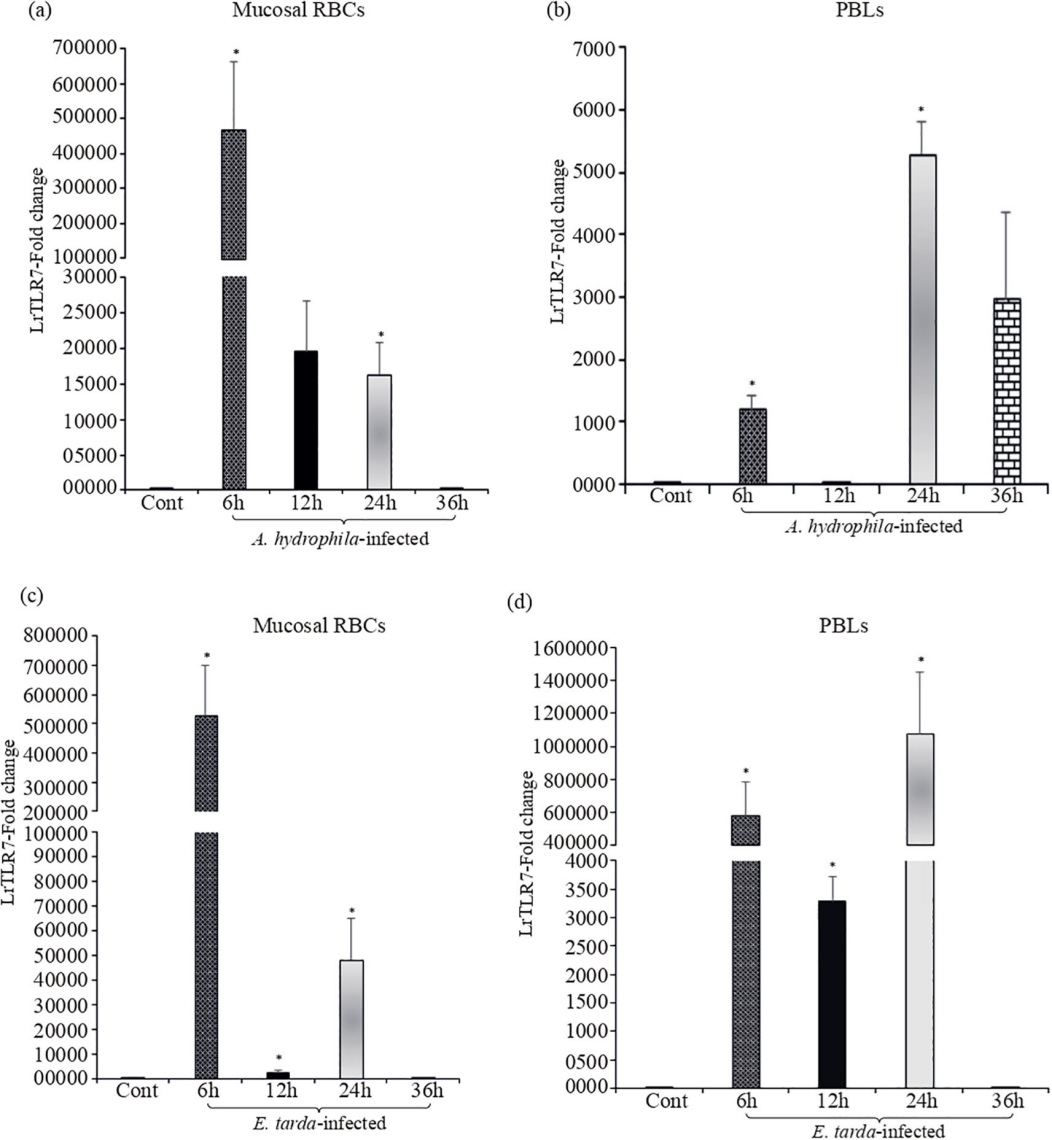
Modulation of LrTLR7 gene expression in RBCs and PBLs following bacterial infections. Rohu fingerlings were either mock-infected (control) or infected with *A*. *hydrophila* or *E*. *tarda* (1×10^6^ CFU fish^−1^) by i.p. injection, and after the designated time course, mucosal RBC or PBLs were isolated from the control and infected fish, total RNA was extracted, and cDNA was prepared. Then, qRT-PCR assay was conducted to analyze the expression of the LrTLR7 gene keeping β-actin as an internal control. Representative data from one experiment out of three separate experiments (N=3) are shown along with the standard error (bars). Significant differences between the control and infected fish RBCs and PBLs are indicated with asterisks (*). LrTLR7 gene expression in the *A*. *hydrophila* infected fish mucosal RBCs **(a)**, and PBLs **(b)**, and *E*. *tarda* infected fish mucosal RBCs **(c)** and PBLs **(d)**.

To further investigate the LrTLR7 gene expression against *A. hydrophila* and *E. tarda* infection at the cellular level, mucosal RBCs and PBLs were isolated from the control and infected fish gills and blood, followed by qRT-PCR analysis of the LrTLR7 gene expression. At 6 h.p.i, LrTLR7 gene expression was ~472,514- fold and ~527,934-fold in the RBCs of *A. hydrophila*- and *E. tarda*-infected fish respectively (**Figures 6a, c**). In the PBLs, enhanced expression of LrTLR7 gene was observed in *A. hydrophila*-infected (6 h, ~1,209-fold, 24 h, ~5,293-fold, 36 h, ~2,977-fold) and *E. tarda-*infected (6 h, ~581,733 fold, 24 h, ~1,078,055 fold) (**Figures 6b, d**).

### 3.4 Response of the LrTLR7 gene during bacterial pathogenesis

**Figure 7:**
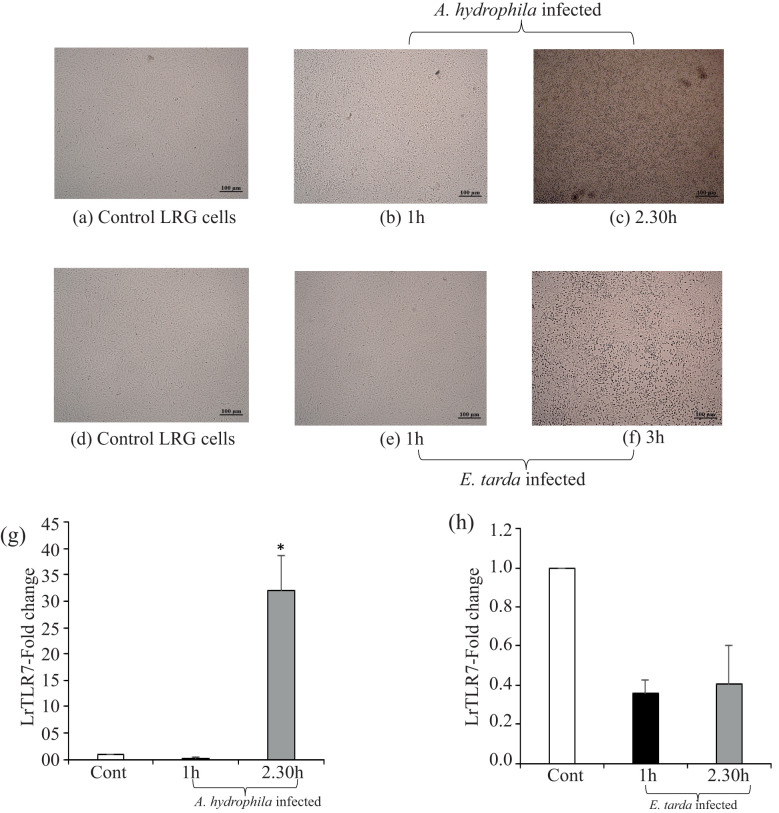
LrTLR7 is induced in LRG cells during bacterial pathogenesis. The *L. rohita* gill (LRG) cell line was cultured for 48 h, and then it was either mock-infected or infected with *A*. *hydrophila* or *E*. *tarda* (with 1 MOI) for the respective time points. Then, the control and infected cells were observed under microscope (10×) to visualize the morphological changes following infections. After the designated time, total RNA was extracted from the mock-infected and bacteria infected cells, and cDNA was synthesized followed by qRT-PCR assay to analyze the LrTLR7 gene expression keeping β-actin as an internal control. The results are expressed as one representative data from three separate experiments (N=3) along with standard error (bars). Significant (p< 0.05) differences between the control and infected cells are indicated with asterisks (*). **(a)** Control, **(b)** 1 h-*A. hydrophila-*infected, **(c)** 2.30 h-*A. hydrophila-*infected, **(d)** control, **(e)** 1 h-*E. tarda-*infected, **(f)** 3 h-*E. tarda-*infected LRG cells. LrTLR7 gene expression post *A*. *hydrophila* **(g)** and *E. tarda* **(h)** infection.

To investigate the response of the LrTLR7 gene during the progression of the bacterial infections, LRG cells were cultured *in-vitro* and were infected with *A. hydrophila* or *E. tarda* for various time courses. In the uninfected-control LRG cells, there were no morphological changes (**Figures 7a, d**), but in the infected LRG cells, a cytopathic effect (CPE) with the shrinkage, clustering, and detachment of cells was observed. Pronounced CPE was detected at 1–2.30 h (**Figures 7b, c**) in the *A. hydrophila*-infected LRG cells, and at 1–3 h post-infection in the *E. tarda*-infected cells (**Figures 7e, f**). The qRT-PCR analysis of the LrTLR7 gene expression in the infected LRG cells revealed ~32-fold enhancement of the LrTLR7 gene at 2.30 h post *A. hydrophila* infection (**Figure 7g**) while it is recorded to be down-regulated in *E. tarda* infection (**Figure 7h**).

### 3.5 Modulation of LrTLR7 gene expression in response to PAMP stimulation

#### 3.5.1 *In-vivo* modulation of LrTLR7 gene expression

**Figure 8:**
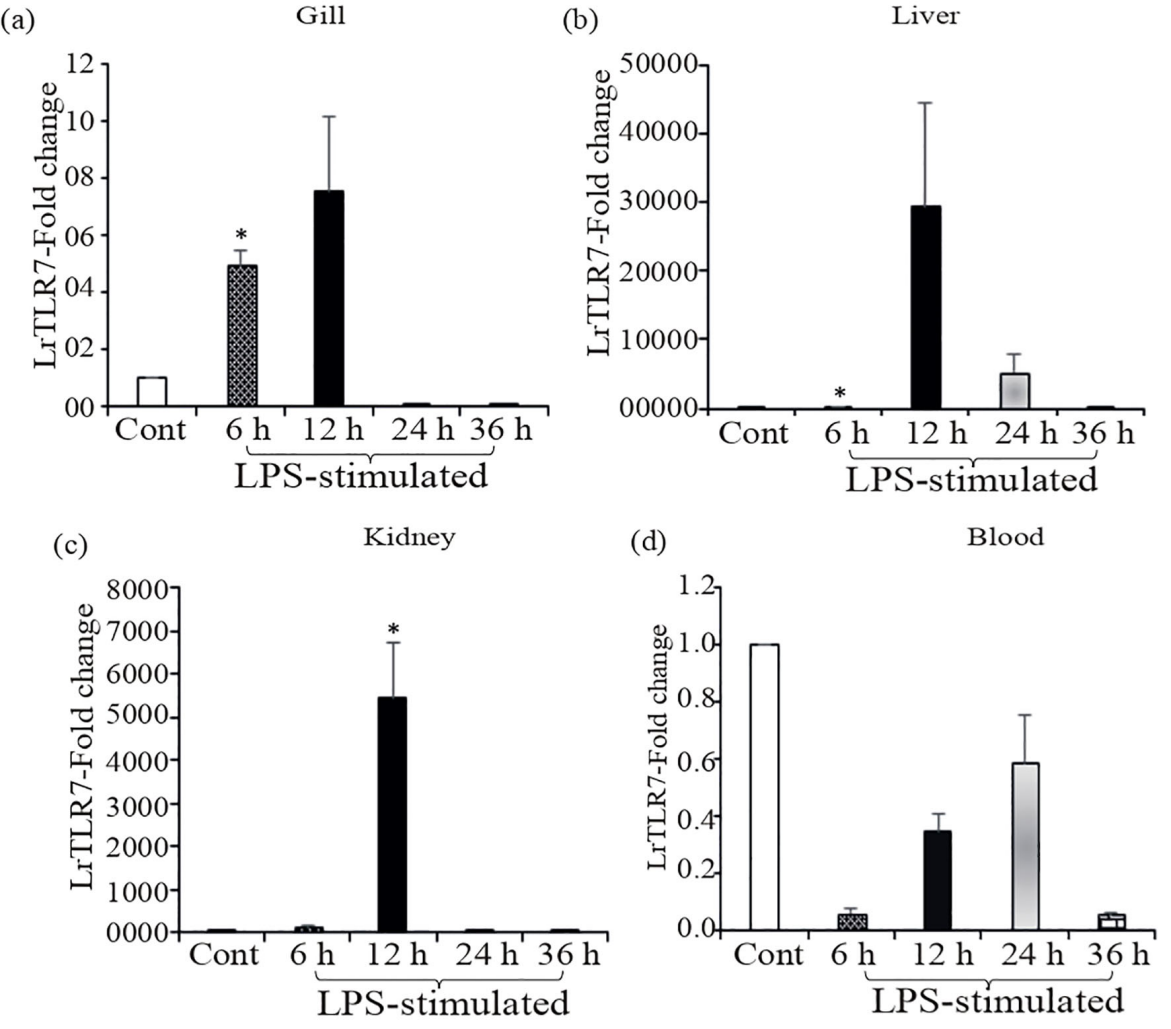
*In-vivo* induction of LrTLR7 gene in response to LPS stimulation. Rohu fingerlings were either mock-stimulated or i.p. injected with LPS (50 µg/fish), and following the time course, total RNA was extracted from the control and LPS-stimulated fish tissues at 6, 12, 24, and 36 h post-stimulation. The cDNA was prepared, and the LrTLR7 gene expression was analyzed through qRT-PCR assay keeping β-actin as an internal control. The results are expressed as one representative data from three separate experiments (N=3) along with standard error (bars). Significant (p<0.05) differences between the control and LPS-stimulated samples are indicated with asterisks (*). LrTLR7 gene expression in the LPS-stimulated fish group: **(a)** gill, **(b)** liver, **(c)** kidney, and **(d)** blood.

Compared with the control, in the LPS-stimulated fish tissues, significant (p<0.05) induction of LrTLR7 gene expression was observed at 6, 12, and 24 h post-stimulation. In the gill, LrTLR7 gene expression was ~5-fold at 6 h and ~7-fold at 12 h (**Figure 8a**), and in the liver, it was ~42-fold at 6 h, ~29,532-fold at 12 h and ~5,404 fold at 24-h post-stimulation (**Figure 8b**). Similarly, in the kidney, LrTLR7 gene expression was ~124-fold at 6 h, and ~5,442-fold at 12 h (**Figure 8c**). In contrast to these tissues, LrTLR7 gene expression was downregulated in the blood (**Figure 8d**).

**Figure 9:**
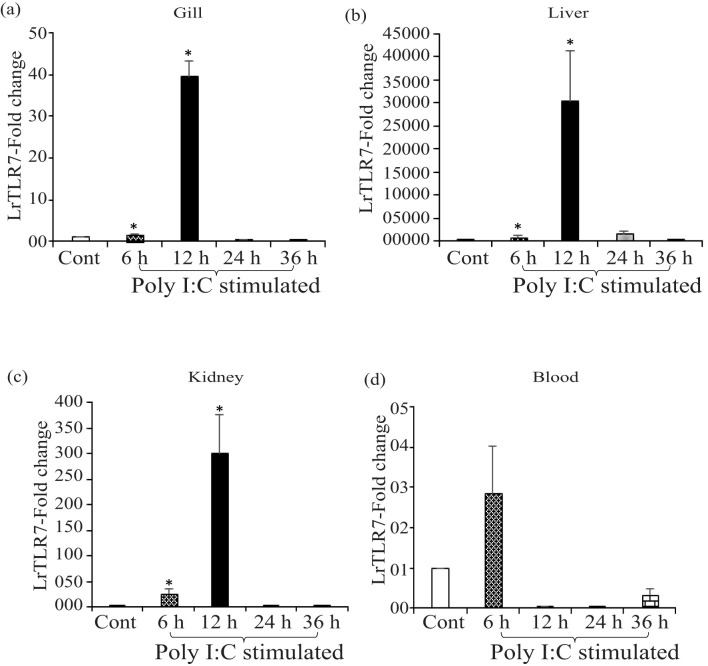
*In-vivo* induction of LrTLR7 gene in response to poly I:C stimulation. Rohu fingerlings were either mock-stimulated or i.p. injected with poly I:C (200 µg/fish), and following the time course, total RNA was extracted from the control and stimulated fish tissues at 6, 12, 24, and 36 h post-stimulation. The cDNA was prepared, and the LrTLR7 gene expression was analyzed through qRT-PCR assay, keeping β-actin as an internal control. The results are expressed as one representative data from three separate experiments (N=3) along with standard error (bars). Significant (p<0.05) differences between the control and stimulated samples are indicated with asterisks (*). LrTLR7 gene expression in the poly I:C-stimulated fish group: **(a)** gill, **(b)** liver, **(c)** kidney, and **(d)** blood.

In the poly I:C stimulation, a similar trend of inductive expression of the LrTLR7 gene was observed in various tissues. In the gill, the expression of LrTLR7 was significantly enhanced (~39-fold) at 12 h post-stimulation (**Figure 9a**). Among all tested tissues, the highest (~30,573-fold) expression of the LrTLR7 gene was recorded in the liver at 12 h (**Figure 9b**), followed by the kidney (~300-fold) at the same time point (**Figure 9c**). In the blood, LrTLR7 was moderately (~2-fold) induced at 6 h post-stimulation (**Figure 9d**).

#### 3.5.2 *In-vitro* modulation of LrTLR7 gene expression

For the confirmation of the *in-vivo* data of LrTLR7 gene induction in response to PAMP stimulation, *in-vitro* analysis of LrTLR7 gene expression was conducted in the purified mucosal RBCs and PBLs following LPS and poly I:C stimulation. Density gradient centrifugation with HiSep™ LSM 1077 followed by Giemsa staining and microscopical observations (100×) of the purified cells revealed a homogenous population of cells with the characteristic features of RBCs and PBLs (**Supplementary Figure S1**).

**Figure 10:**
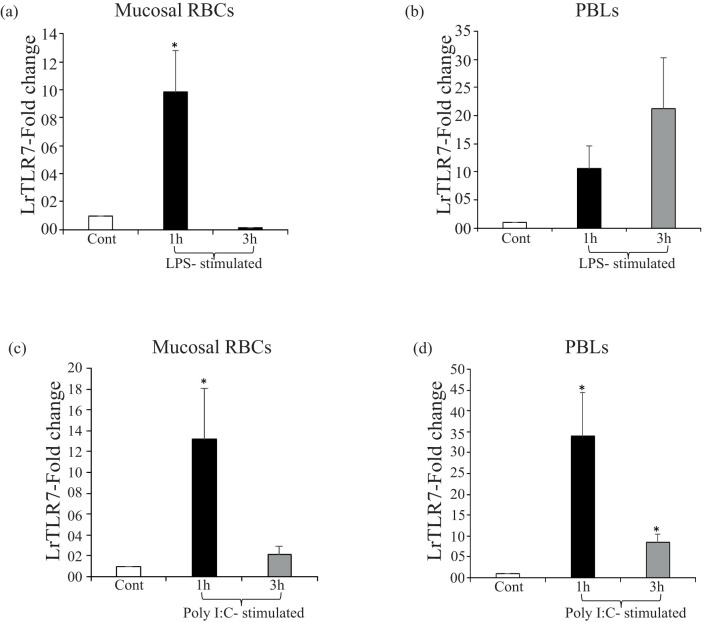
*In-vitro* modulation of LrTLR7 gene expression in the mucosal RBCs and PBLs following LPS and poly I:C stimulation. Purified mucosal RBCs and PBLs (10^7^ cells/ml^/^well) were cultured in their respective media and were either mock-stimulated or stimulated with LPS (in RBCs: 10 µg/ml/well and in PBLs: 20 µg/ml^/^well) or poly I:C (RBCs and PBLs: 100 µg/ml/well) for 1 and 3 h. After the designated time course, total RNA was extracted from the control and LPS or poly I:C-stimulated cells and cDNA was prepared. The qRT-PCR assay was conducted to analyze the LrTLR7 gene expression, keeping β-actin as an internal control. Representative data of one experiment out of three separate experiments (N=3) are shown along with the standard error (bars). A significant (p<0.05) difference in LrTLR7 gene expression between the control and stimulated RBCs and PBLs has been indicated with asterisks (*). LPS-stimulated mucosal RBCs **(a)** and PBLs **(b)**; and poly I:C-stimulated mucosal RBCs **(c)** and PBLs **(d)**.

LrTLR7 gene expression in the purified mucosal RBCs was induced by ~9-fold at 1 h following LPS stimulation (**Figure 10a**), whereas poly I:C stimulation resulted in the induction of LrTLR7 gene expression by ~13-fold at 1 h and ~2-fold at 3 h (**Figure 10c**). In the PBLs, LrTLR7 gene expression was induced by both LPS stimulation (1 h, ~10-fold, and 3 h, ~21-fold) (**Figure 10b**) and poly I:C stimulation (1 h, ~34-fold, and 3 h, ~8-fold)] (**Figure 10d**).

### 3.6 Analysis of the LrTLR7 signal transduction pathway

**Figure 11:**
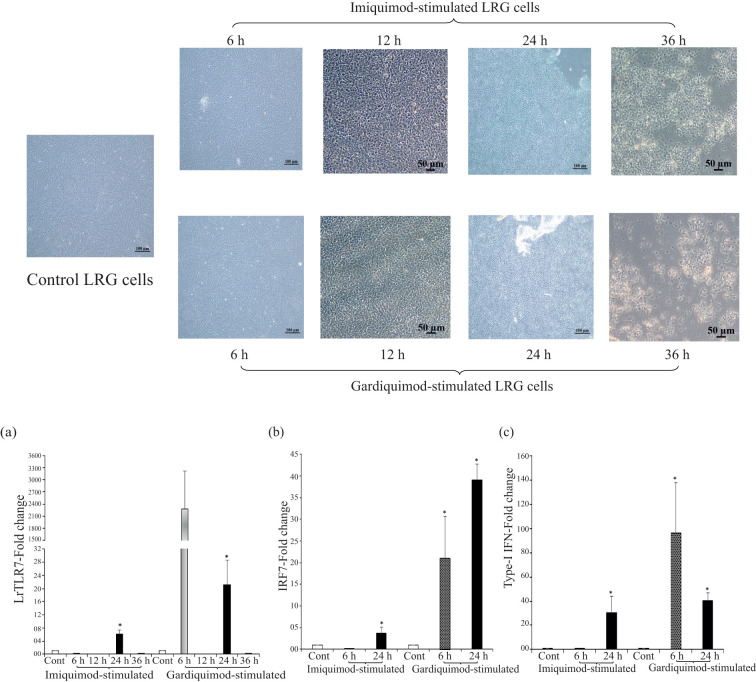
*In-vitro* activation of LrTLR7-signal transduction pathways by imiquimod and gardiquimod. The *L. rohita* gill (LRG) cell line was cultured for 48 h and was either mock-stimulated or stimulated with imiquimod or gardiquimod (10 μg/ml), and then the morphological changes of the cells were observed under microscope (10×) (upper panel figures). After the designated time course, total RNA was extracted from the mock-stimulated and ligand-stimulated cells followed by qRT-PCR assay to analyze the LrTLR7, IRF7, and type-I IFN gene expression, keeping β-actin as an internal control. The results are expressed as one representative data from three separate experiments (N=3) along with the standard error (bars). Significant (p<0.05) differences between the control and stimulated LRG cells are indicated with asterisks (*). **(a)** LrTLR7 gene expression, **(b)** IRF7 gene expression, and **(c)** type-I IFN gene expression in the imiquimod and gardiquimod stimulated LRG cells.

To analyze the LrTLR7 signal transduction pathways, LRG cells were either mock-stimulated or stimulated with imiquimod and gardiquimod [known TLR7 ligands in the higher vertebrates (39)] and the expression of the LrTLR7 gene was analyzed at 6, 12, 24, and 36 h post-stimulation. Till 24 h post-stimulation, both mock-stimulated (control) cells and imiquimod- and gardiquimod-stimulated cells did not have any significant morphological changes, but at 36 h post-stimulation, all cells (control and stimulated) started to undergo apoptosis showing the feature of shrinkage, cluster formation, and detachment from the plate surface (**Figure 11**, upper panel).

In comparison with the unstimulated control, the LrTLR7 gene expression in the imiquimod-stimulated LRG cells was significantly (p<0.05) elevated ~6-fold at 24 h. On gardiquimod stimulation, the expression of LrTLR7 was also upregulated ~2,288-fold at 6 h, and with the advancement of time, it was reduced to ~21-fold at 24 h (**Figure 11a**).

To further analyze the LrTLR7-signal transduction pathways, the expression of IRF7, a downstream adaptor molecule, and type-I IFN, the effector molecule was also analyzed in the LRG cells post imiquimod and gardiquimod stimulation. Compared with the control, at 24 h post-stimulation, the IRF7 was enhanced to ~3-fold following imiquimod stimulation and ~39-fold following gardiquimod stimulation (**Figure 11b**). Similarly, Type-I IFN gene expression was enhanced at 24 h post-stimulation by ~30-fold and ~40-fold, respectively, following imiquimod and gardiquimod stimulation (**Figure 11c**).

**Figure 12:**
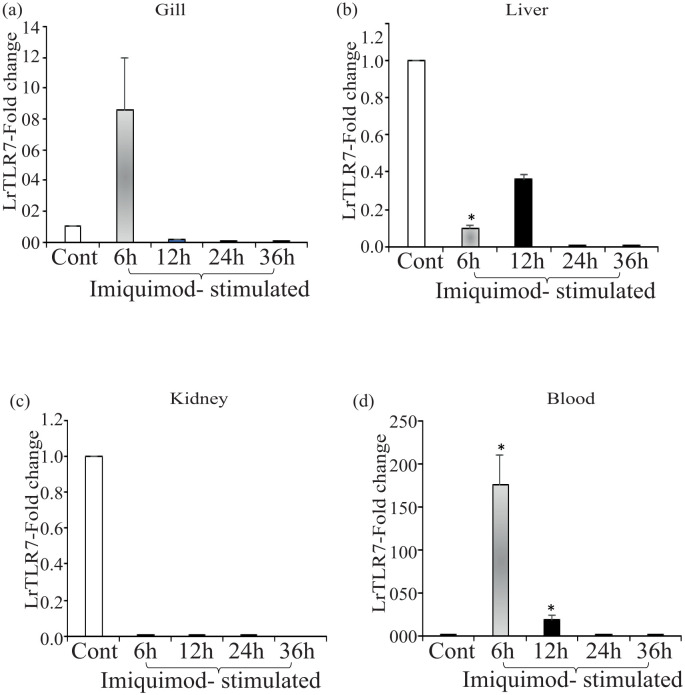
*In-vivo* LrTLR7 gene expression in response to imiquimod stimulation. Rohu fingerlings were either mock-stimulated or i.p. injected with imiquimod (10 µg/fish), and following the time course, total RNA was extracted from the control and stimulated fish tissues. The cDNA was prepared, and LrTLR7 gene expression was analyzed through qRT-PCR assay keeping β-actin as an internal control. The results are expressed as one representative data from three separate experiments (N=3) along with the standard error (bars). Significant (p<0.05) differences between the control and imiquimod-stimulated fish tissues have been indicated with asterisks (*). LrTLR7 gene expression in the imiquimod-stimulated fish group: **(a)** gill, **(b)** liver, **(c)** kidney, and **(d)** blood.

**Figure 13:**
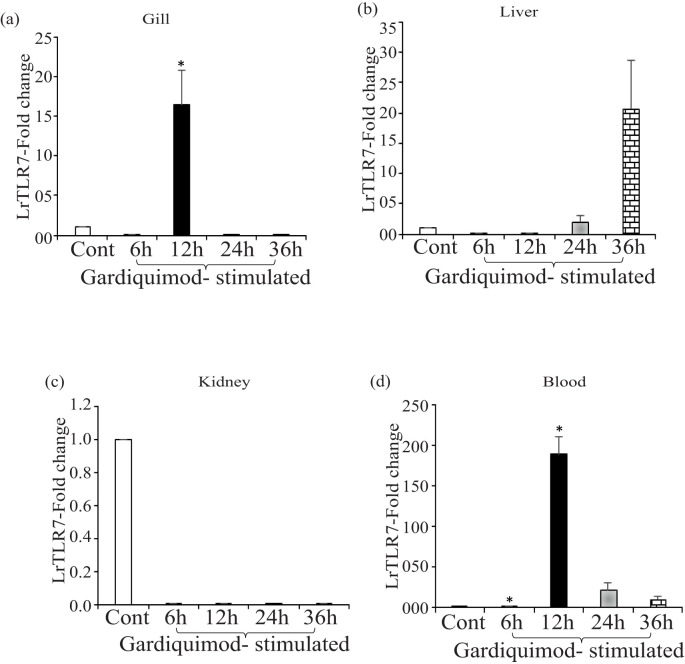
*In-vivo* LrTLR7 gene expression in response to gardiquimod stimulation. Rohu fingerlings were either mock-stimulated or i.p. injected with gardiquimod (10 µg/fish), and following the time course, total RNA was extracted from the control and stimulated fish tissues at 6, 12, 24, and 36 h post-stimulation. The cDNA was prepared, and LrTLR7 gene expression was analyzed through qRT-PCR assay keeping β-actin as an internal control. The results are expressed as one representative data from three separate experiments (N=3) along with the standard error (bars). Significant (p<0.05) differences between the control and gardiquimod-stimulated fish tissues have been indicated with asterisks (*). LrTLR7 gene expression in the gardiquimod-stimulated fish group: **(a)** gill, **(b)** liver, **(c)** kidney, and **(d)** blood.

Next, we checked the LrTLR7 signal transduction pathways in the *L. rohita* fingerlings following stimulation with the imiquimod and gardiquimod. *In-vivo* stimulation with imiquimod resulted in significantly (p<0.05) enhanced LrTLR7 gene expression in gill (6 h, ~8-fold) (**Figure 12a**) and blood (6 h, ~176-fold; 12 h, ~18-fold) (**Figure 12d**) while it is downregulated in kidney and liver (**Figures 12b, c**). In gardiquimod stimulation, except in the kidney, while it is down-regulated in kidney and liver (**Figures 12b, c**), elevated LrTLR7 gene expression was observed in the gill (12 h, ~16-fold) (**Figure 13a**), liver (24 h, ~2-fold; 36 h, ~20-fold) (**Figure 13b**), and blood (12 h, ~190-fold, 24 h, ~22-fold, and 36h, ~11-fold) (**Figure 13d**), but it was down-regulated in kidney (**Figure 13c**).

**Figure 14:**
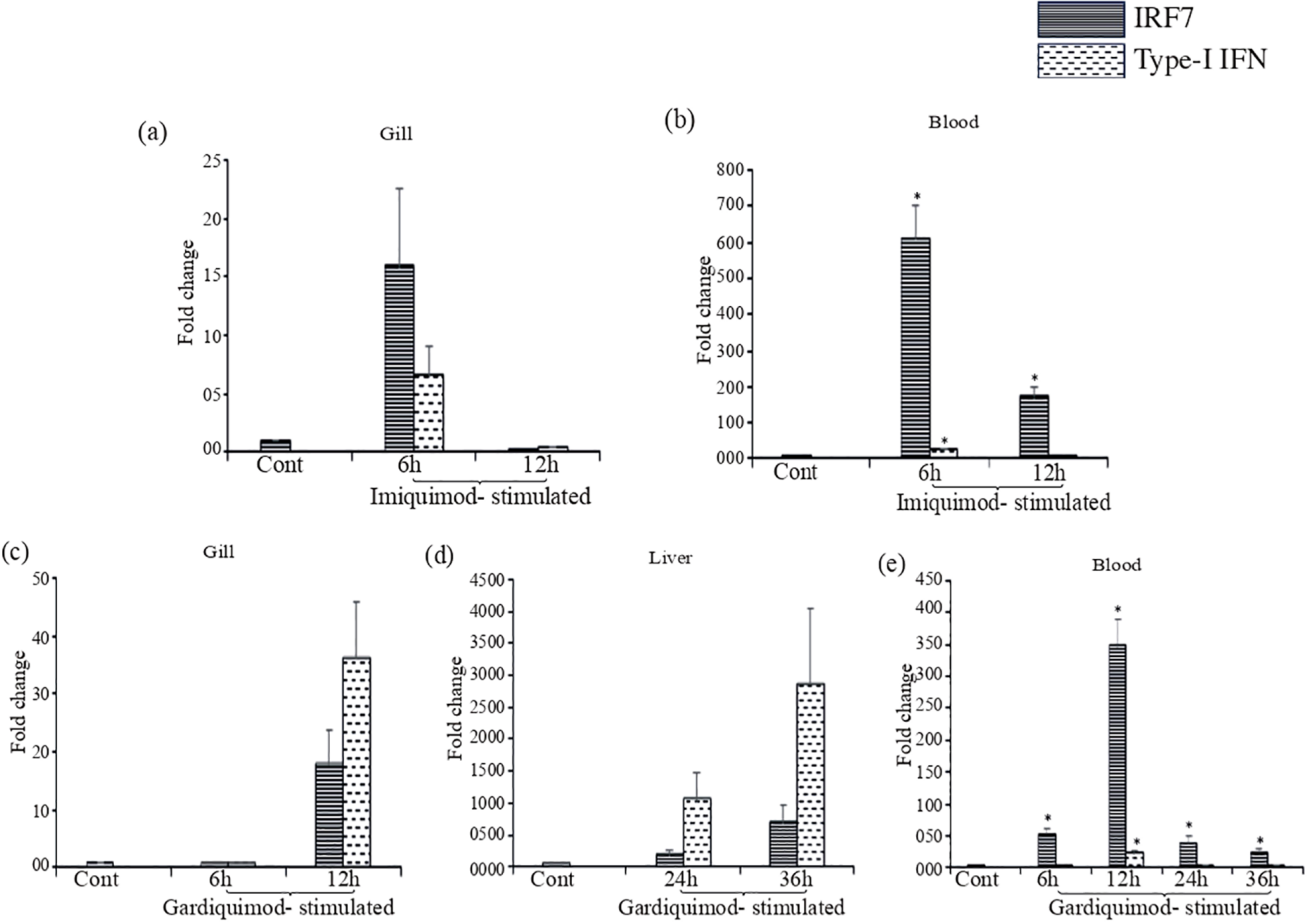
IRF7 and type I IFN gene expression in response to imiquimod and gardiquimod stimulation. Rohu fingerlings were either mock-stimulated or i.p. injected with imiquimod or gardiquimod and following the time course, total RNA was extracted from the control and stimulated fish tissues. The cDNA was prepared and expression of IRF7, and type-I IFN genes were analyzed through qRT-PCR assay keeping β-actin as an internal control. The results are expressed as one representative data from three separate experiments (N=3) along with standard error (bars). Significant (p<0.05) differences between the control and stimulated fish samples have been indicated with asterisks (*). In imiquimod-stimulated fish tissues, IRF7 gene expression and type- I IFN gene expression are shown in gill **(a)** and blood **(b)**. In gardiquimod-stimulated fish tissues, IRF7 gene expression and type I IFN gene expression are shown in gill **(c)**, liver **(d)**, and blood **(e)**.

To analyze the imiquimod- and gardiquimod-mediated *in-vivo* TLR7-signal transduction pathway, the expression profile of IRF7 and Type-I IFN gene expression was also investigated in the same tissues (gill and blood), where LrTLR7 gene expression was enhanced. The result revealed that following imiquimod stimulation, there was a good correlation of LrTLR7 in the gill (**Figure 13a**), along with the IRF7 and Type-I IFN (**Figure 14a**) and also LrTLR7 in the blood (**Figure 13d**) with IRF7 and Type-I IFN (**Figure 14b**). In the gardiquimod-stimulated fish gill, liver, and blood, a similar trend of enhanced LrTLR7 (**Figures 13a, b, d**) with IRF7, and type-I IFN gene expression (**Figures 14c–e**) was observed suggesting the activation of the LrTLR7-signal transduction pathway.

## 4 Discussion

This article describes the molecular cloning and characterization of TLR7 in *L. rohita*, a prime freshwater fish species in Southeast Asian countries. LrTLR7 was structurally more similar to TLR7 proteins of other fish species than higher vertebrates. In the phylogenetic analysis, LrTLR7 was closely associated with TLR7 proteins of the *Cyprinidae* family.

Innate immune genes are expected to play a key role against pathogenic invasions, especially during the embryonic developmental stages. Expression of the LrTLR7 gene in various developmental stages suggests its important role in protecting embryos against infections. This is the first study to elucidate the expression of LrTLR7 in various developmental stages of a fish and is expected to serve as a reference for future studies. The LrTLR7 gene was expressed in most of the examined tissues in rohu fingerlings, with a prominent expression observed in the gills. Similar expression profiles have been observed in other fish species such as large yellow croaker and spiny eel (16, 40).

Previous studies have shown that TLR7 is upregulated in various tissues of tongue sole and spotted sea bass upon infection with Gram-negative bacteria, specifically *Pseudomonas fluorescence* and *Vibrio harveyi*, respectively (26, 41). Similarly, infection of rohu fingerlings with *A. hydrophila* or *E. tarda* also resulted in significantly enhanced expression of the LrTLR7 gene in the liver, kidney, and blood tissues, but in the gill tissues, it was downregulated. On the other hand, mucosal RBCs purified from the filaments of the same infected fish gill exhibited a significantly very high expression of the LrTLR7 gene as compared with the uninfected control fish. This contrasting data may be due to the fact that the gill tissues composed of filaments, rakers, and arches, and within this, mucosal RBCs in the gill filaments share only a very small component. Therefore, while analyzing the LrTLR7 gene expression in the whole gill tissue, the contribution of a very small number of mucosal RBCs in expressing LrTLR7 gene were perhaps masked by the other greater number of cell types and tissues, resulting in downregulation of the LrTLR7 gene. In the isolated and purified greater number of mucosal RBCs, this masking effect was possibly eliminated resulting in remarkably very high expression of LrTLR7 gene. However, further detailed investigation is needed to confirm it.

To identify the specific component of the Gram-negative bacteria that may trigger LrTLR7 expression, we focused on LPS, a major constituent in their outer membrane. Although LPS is a known ligand of TLR4, in various teleost species, stimulation with LPS has been shown to induce the expression of a wide range of TLR genes, including TLR2a, TLR2b, TLR5S, TLR7, TLR8, TLR9, TLR13c, TLR14, and TLR22 (42). In rohu, we investigated the response of LrTLR7 to LPS by stimulating rohu fingerlings *in-vivo* and in the RBCs and PBLs *in-vitro*. The expression profile of LrTLR7 closely resembled previous findings, indicating that LrTLR7 also responds to LPS stimulation at tissue and cellular levels.

Fishes are also infected with various viruses having dsRNA as their genetic materials such as viral hemorrhagic septicaemia (VHS) virus. Poly I:C is a synthetic double-stranded RNA that simulates the function of viral dsRNA. In the large yellow croaker (16), golden pompano (18), and *Ctenopharyngodon idella* kidney (CIK) cells, the TLR7 gene was activated by poly I:C (43). To investigate the response of LrTLR7 against dsRNA, poly I:C was injected into the rohu fingerlings and purified RBCs and PBLs were also stimulated with poly I:C *in-vitro*. In both studies, significant upregulation of the LrTLR7 gene expression was observed, suggesting the possible role of TLR7 in recognizing dsRNA-viral infection.

Studies have shown that imidazoquinoline compounds are capable of activating TLR7 expression (44, 45). In the Atlantic salmon (*S. salar*), the antiviral effects in the liver and head kidney were triggered by the imiquimod derivative S-27609 via the TLR7–MyD88-dependent signaling pathway (46). In common carp, TLR7 activation occurred in the head kidney cells following imiquimod administration, leading to the induction of Type-I IFN expression (14). Consistent with these findings, *in-vitro* stimulation of LRG cells and *in-vivo* stimulation of the rohu fingerlings with the imiquimod and gardiquimod, also resulted in the significant (p<0.05) induction of LrTLR7. Furthermore, in the LrTLR7-expressing tissues, a very good correlation was observed in the enhanced expression pattern of LrTLR7, its downstream molecule IRF7, and the effector molecule type-I IFN. Overall, these data indicate that, like higher vertebrates, LrTLR7 in rohu may play a critical immune role against diverse pathogenic invasions.

## 5 Conclusion

Like higher vertebrates, TLR7 in fish responds to viruses and their ss/ds-stranded RNA and also induces broadly acting innate immunity against some bacterial pathogens. Several TLR7 agonists have been reported to induce innate immune responses in many mammals, including fish. In this article, we have shown that LrTLR7 responds against LPS, poly I:C, imiquimod, and gardiquimod, and also against two most important fish pathogens such as *A. hydrophila* and *E. tarda*. These data suggest that TLR7 can be targeted using specific agonists or ligands that interact with and activate it, functioning as an immunoadjuvant as well as a trained immunity-based vaccine (TibV) to prevent diseases in fish.

## Data Availability

The datasets presented in this study can be found in online repositories. The names of the repository/repositories and accession number(s) can be found below: https://www.ncbi.nlm.nih.gov/genbank/, PP760378.

## Supplementary material

Supplementary Figure 1 Purified red blood cells (RBCs) and peripheral blood leukocytes (PBLs) of *Labeo rohita.* Mucosal RBCs and PBLs were isolated and purified from the *L. rohita* fingerlings following density gradient centrifugation. Subsequently, they were observed under microscope without staining (20X) or staining with Giemsa under the oil immersion lens (100X). Unstained RBCs **(a)** and Giemsa-stained RBCs **(b)**; unstained PBLs **(c)** and Giemsa-stained PBLs **(d)**.

## References

[1] Akira S Uematsu S Takeuchi O. Pathogen recognition and innate immunity. Cell. (2006) 124:783–801. doi: 10.1016/j.cell.2006.02.015 16497588

[2] Rauta PR Samanta M Dash HR Nayak B Das S. Toll-like receptors (TLRs) in aquatic animals: signaling pathways, expressions and immune responses. Immunol Lett. (2014) 158:14–24. doi: 10.1016/j.imlet.2013.11.013 24291116

[3] Kawai T Akira S. The role of pattern-recognition receptors in innate immunity: update on Toll-like receptors. Nat Immunol. (2010) 11:373–84. doi: 10.1038/ni.1863 20404851

[4] Takeda K Akira S. Toll-like receptors. Curr Protoc Immunol. (2015) 109:14–2. doi: 10.1002/0471142735.im1412s109 25845562

[5] Lim KH Staudt LM. Toll-like receptor signaling. Cold Spring Harbor Perspect Biol. (2013) 5:a011247. doi: 10.1101/cshperspect.a011247 PMC3579400 23284045

[6] Iliev DB Roach JC Mackenzie S Planas JV Goetz FW. Endotoxin recognition: in fish or not in fish? FEBS Lett. (2005) 579:6519–28. doi: 10.1016/j.febslet.2005.10.061 PMC1365396 16297386

[7] Mahapatra S Ganguly B Pani S Saha A Samanta M. A comprehensive review on the dynamic role of toll-like receptors (TLRs) in frontier aquaculture research and as a promising avenue for fish disease management. Int J Biol Macromolecules. (2023) 253:126541. doi: 10.1016/j.ijbiomac.2023.126541 37648127

[8] Heil F Hemmi H Hochrein H Ampenberger F Kirschning C Akira S. Species-specific recognition of single-stranded RNA via toll-like receptor 7 and 8. Science. (2004) 303:1526–9. doi: 10.1126/science.1093620 14976262

[9] Xinxian W Peng J Guixiang T Jinjin W Xiaocong Z Junqiang H. Effect of common carp (Cyprinus carpio) TLR9 overexpression on the expression of downstream interferon-associated immune factor mRNAs in epithelioma papulosum cyprini cells. Veterinary Immunol immunopathology. (2016) 170:47–53. doi: 10.1016/j.vetimm.2015.10.006 26848048

[10] Meijer AH Krens SG Rodriguez IAM He S Bitter W Snaar-Jagalska BE. Expression analysis of the Toll-like receptor and TIR domain adaptor families of zebrafish. Mol Immunol. (2004) 40:773–83. doi: 10.1016/j.molimm.2003.10.003 14687934

[11] Oshiumi H Tsujita T Shida K Matsumoto M Ikeo K Seya T. Prediction of the prototype of the human Toll-like receptor gene family from the pufferfish, Fugu rubripes, genome. Immunogenetics. (2003) 54:791–800. doi: 10.1007/s00251-002-0519-8 12618912

[12] Skjæveland I Iliev DB Strandskog G Jørgensen JB. Identification and characterization of TLR8 and MyD88 homologs in Atlantic salmon (Salmo salar). Dev Comp Immunol. (2009) 33:1011–7. doi: 10.1016/j.dci.2009.04.007 19422846

[13] Palti Y Gahr SA Purcell MK Hadidi S Rexroad CE III Wiens GD. Identification, characterization and genetic mapping of TLR7, TLR8a1 and TLR8a2 genes in rainbow trout (Oncorhynchus mykiss). Dev Comp Immunol. (2010) 34:219–33. doi: 10.1016/j.dci.2009.10.002 19825389

[14] Tanekhy M Kono T Sakai M. Cloning, characterization, and expression analysis of Toll-like receptor-7 cDNA from common carp, Cyprinus carpio L. Comp Biochem Physiol Part D: Genomics Proteomics. (2010) 5:245–55. doi: 10.1016/j.cbd.2010.07.001 20709611

[15] Zhang J Liu S Rajendran KV Sun L Zhang Y Sun F. Pathogen recognition receptors in channel catfish: III Phylogeny and expression analysis of Toll-like receptors. Dev Comp Immunol. (2013) 40:185–94. doi: 10.1016/j.dci.2013.01.009 23396097

[16] Qian T Wang K Mu Y Ao J Chen X. Molecular characterization and expression analysis of TLR 7 and TLR 8 homologs in large yellow croaker (Pseudosciaena crocea). Fish shellfish Immunol. (2013) 35:671–9. doi: 10.1016/j.fsi.2013.05.019 23742866

[17] Dong X Su B Zhou S Shang M Yan H Liu F. Identification and expression analysis of toll-like receptor genes (TLR8 and TLR9) in mucosal tissues of turbot (Scophthalmus maximus L.) following bacterial challenge. Fish Shellfish Immunol. (2016) 58:309–17. doi: 10.1016/j.fsi.2016.09.021 27633670

[18] Wei Y Hu S Sun B Zhang Q Qiao G Wang Z. Molecular cloning and expression analysis of toll-like receptor genes (TLR7, TLR8 and TLR9) of golden pompano (Trachinotus ovatus). Fish Shellfish Immunol. (2017) 63:270–6. doi: 10.1016/j.fsi.2017.02.026 28232281

[19] Mikami T Miyashita H Takatsuka S Kuroki Y Matsushima N. Molecular evolution of vertebrate Toll-like receptors: evolutionary rate difference between their leucine-rich repeats and their TIR domains. Gene. (2012) 503:235–43. doi: 10.1016/j.gene.2012.04.007 22587897

[20] Blasius AL Beutler B. Intracellular toll-like receptors. Immunity. (2010) 32:305–15. doi: 10.1016/j.immuni.2010.03.012 20346772

[21] Zhu LY Nie L Zhu G Xiang LX Shao JZ. Advances in research of fish immune-relevant genes: a comparative overview of innate and adaptive immunity in teleosts. Dev Comp Immunol. (2013) 39:39–62. doi: 10.1016/j.dci.2012.04.001 22504163

[22] Zhang YB Gui JF. Molecular regulation of interferon antiviral response in fish. Dev Comp Immunol. (2012) 38:193–202. doi: 10.1016/j.dci.2012.06.003 22721905

[23] Hemmi H Kaisho T Takeuchi O Sato S Sanjo H Hoshino K. Small anti-viral compounds activate immune cells via the TLR7 MyD88-dependent signaling pathway. Nat Immunol. (2002) 3:196–200. doi: 10.1038/ni758 11812998

[24] Itoh H Tatematsu M Watanabe A Iwano K Funami K Seya T. UNC93B1 physically associates with human TLR8 and regulates TLR8-mediated signaling. PloS One. (2011) 6:e28500. doi: 10.1371/journal.pone.0028500 22164301 PMC3229609

[25] Kim YM Brinkmann MM Paquet ME Ploegh HL. UNC93B1 delivers nucleotide-sensing toll-like receptors to endolysosomes. Nature. (2008) 452:234–8. doi: 10.1038/nature06726 18305481

[26] Li XP Sun L. TLR7 is required for optimal immune defense against bacterial infection in tongue sole (Cynoglossus semilaevis). Fish Shellfish Immunol. (2015) 47:93–9. doi: 10.1016/j.fsi.2015.08.025 26327112

[27] FAO. Labeo rohita. In: Cultured aquatic species fact sheets (2009) (Rome, Italy: FAO).

[28] Barde RD. Bacterial diversity associated with Labeo rohita isolated from cultured and natural water bodies. Eur J Mol Clin Med. (2020) 7(11):2020.

[29] Nhinh DT Le DV Van KV Huong Giang NT Dang LT Hoai TD. Prevalence, virulence gene distribution and alarming the multidrug resistance of aeromonas hydrophila associated with disease outbreaks in freshwater aquaculture. Antibiotics. (2021) 10:Article 5. doi: 10.3390/antibiotics10050532 PMC8147934 34064504

[30] Rasmussen-Ivey CR Hossain MJ Odom SE Terhune JS Hemstreet WG Shoemaker CA. Classification of a hypervirulent aeromonas hydrophila pathotype responsible for epidemic outbreaks in warm-water fishes. Front Microbiol. (2016) 7. doi: 10.3389/fmicb.2016.01615 PMC5067525 27803692

[31] Hassan HA Ding X Zhang X Zhu G. Fish borne *Edwardsiella tarda* eha involved in the bacterial biofilm formation, hemolytic activity, adhesion capability and pathogenicity. Arch Microbiol. (2020) 202:835–42. doi: 10.1007/s00203-019-01794-x 31865430

[32] Pandey V Hussain Bhat RA Chandra S Tandel RS Dubey MK Sharma P. Clinical signs, lethal dose and histopathological lesions in grass carp, *Ctenopharyngodon idella* experimentally infected with *Edwardsiella tarda* . Microbial Pathogenesis. (2021) 161:105292. doi: 10.1016/j.micpath.2021.105292 34800633

[33] Thakur K Sharma A Sharma D Brar B Choudhary K Sharma AK. An insight into the interaction between *Argulu*s *siamensis* and *Labeo rohita* offers future therapeutic strategy to combat argulosis. Aquacult Int. (2023) 31:1607–21. doi: 10.1007/s10499-022-01043-x PMC9792311 36589529

[34] Pradhan PK Paria A Yadav MK Verma DK Gupta S Swaminathan TR. Susceptibility of Indian major carp *Labeo rohita* to tilapia lake virus. Aquaculture. (2020) 515:734567. doi: 10.1016/j.aquaculture.2019.734567

[35] Mahapatra S Ganguly B Pani S Saha A Samanta M. RBCs of Labeo rohita express TLRs, NLRs, interleukins, and interferon (IFN)-I in response to Gram-negative bacterial infections and LPS-stimulations. J Fish Biol. (2023) 103:496–506. doi: 10.1111/jfb.15465 37255266

[36] Basavaraju Y Varghese TJ. Embryonic and larval development of rohu-mrigal hybrid. Proceedings. Anim Sci. (1981) 90:417–26. doi: 10.1007/BF03186019

[37] Abdul Majeed S Nambi KSN Taju G Sundar Raj N Madan N Sahul Hameed AS. Establishment and characterization of permanent cell line from gill tissue of Labeo rohita (Hamilton) and its application in gene expression and toxicology. Cell Biol Toxicol. (2013) 29:59–73. doi: 10.1007/s10565-012-9237-7 23224722

[38] Livak KJ Schmittgen TD. Analysis of relative gene expression data using real-time quantitative PCR and the 2(-Delta Delta C(T)) Method. Methods (San Diego Calif.). (2001) 25:402–8. doi: 10.1006/meth.2001.1262 11846609

[39] Meyer T Stockfleth E. Clinical investigations of Toll-like receptor agonists. Expert Opin investigational Drugs. (2008) 17:1051–65. doi: 10.1517/13543784.17.7.1051 18549341

[40] Han C Li Q Liu J Hao Z Huang J Zhang Y. Characterization, evolution, and expression analysis of TLR7 gene subfamily members in Mastacembelus armatus (Synbranchiformes: Mastacembelidae). Dev Comp Immunol. (2019) 95:77–88. doi: 10.1016/j.dci.2019.02.002 30742850

[41] Fan H Wang L Wen H Wang K Qi X Li J. Genome-wide identification and characterization of toll-like receptor genes in spotted sea bass (Lateolabrax maculatus) and their involvement in the host immune response to Vibrio harveyi infection. Fish Shellfish Immunol. (2019) 92:782–91. doi: 10.1016/j.fsi.2019.07.010 31288100

[42] Wang KL Chen SN Huo HJ Nie P. Identification and expression analysis of sixteen Toll-like receptor genes, TLR1, TLR2a, TLR2b, TLR3, TLR5M, TLR5S, TLR7– 9, TLR13a– c, TLR14, TLR21– 23 in mandarin fish Siniperca chuatsi. Dev Comp Immunol. (2021) 121:104100. doi: 10.1016/j.dci.2021.104100 33862097

[43] Yang C Su J Zhang R Peng L Li Q. Identification and expression profiles of grass carp Ctenopharyngodon idella tlr7 in responses to double-stranded RNA and virus infection. J Fish Biol. (2012) 80:2605–22. doi: 10.1111/j.1095-8649.2012.03316.x 22650436

[44] Lee PT Zou J Holland JW Martin SA Kanellos T Secombes CJ. Identification and characterization of TLR7, TLR8a2, TLR8b1 and TLR8b2 genes in Atlantic salmon (Salmo salar). Dev Comp Immunol. (2013) 41:295–305. doi: 10.1016/j.dci.2013.05.013 23747412

[45] Kawai T Akira S. Toll-like receptors and their crosstalk with other innate receptors in infection and immunity. Immunity. (2011) 34:637–50. doi: 10.1016/j.immuni.2011.05.006 21616434

[46] Kileng Ø Albuquerque A Robertsen B. Induction of interferon system genes in Atlantic salmon by the imidazoquinoline S-27609, a ligand for Toll-like receptor 7. Fish shellfish Immunol. (2008) 24:514–22. doi: 10.1016/j.fsi.2007.10.005 18337121

