# Supplementary figures and images for: Molecular characterization and immune role of TLR7 in Labeo rohita
Source: Front Immunol. 2025 Apr 25;16:1555048. doi: 10.3389/fimmu.2025.1555048 (PMC12062079; doi:10.3389/fimmu.2025.1555048)

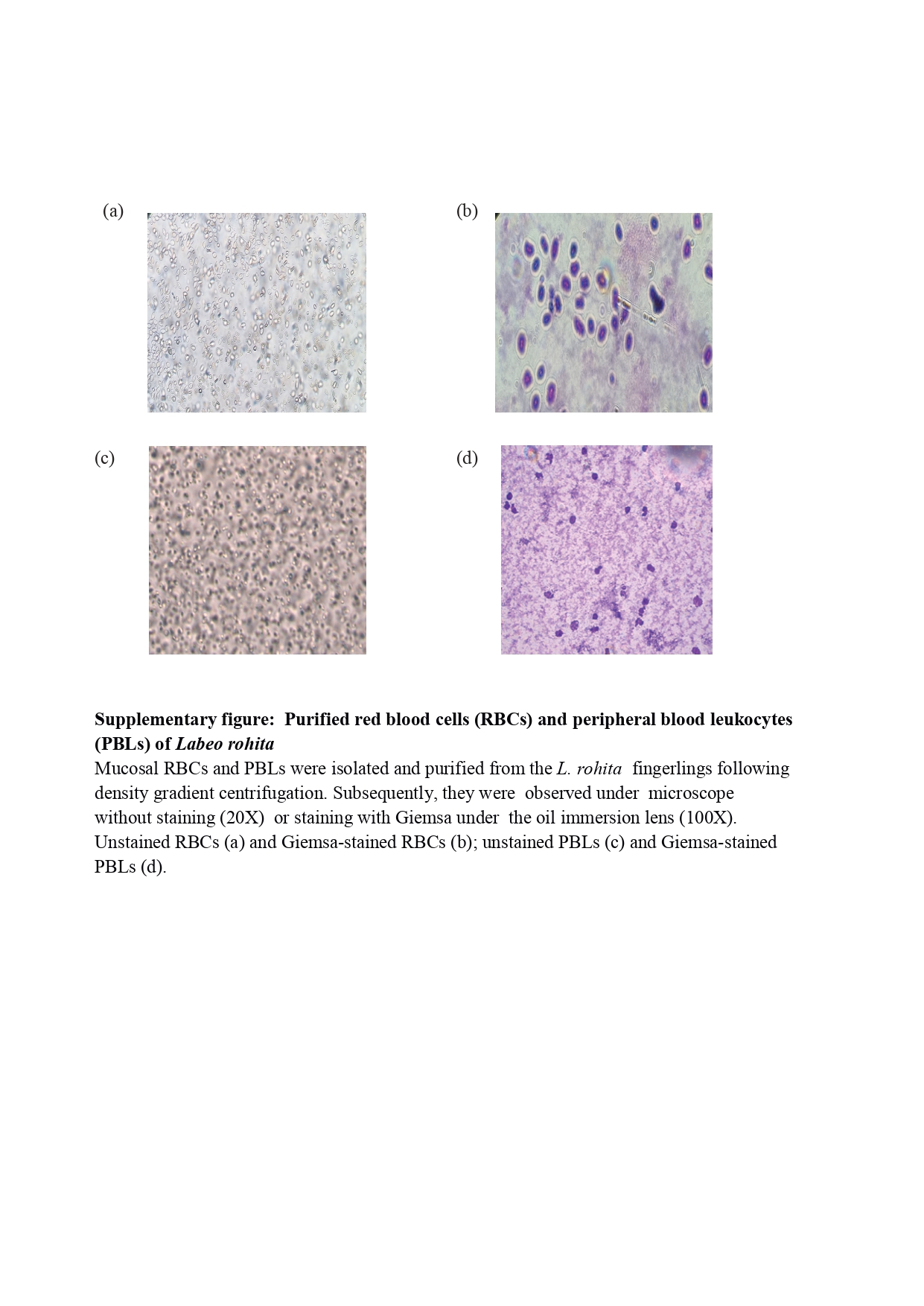

Supplement: Supplementary Figure 1 — Purified red blood cells (RBCs) and peripheral blood leukocytes (PBLs) of Labeo rohita. Mucosal RBCs and PBLs were isolated and purified from the L. rohita fingerlings following density gradient centrifugation. Subsequently, they were observed under microscope without staining (20X) or staining with Giemsa under the oil immersion lens (100X). Unstained RBCs (a) and Giemsa-stained RBCs (b); unstained PBLs (c) and Giemsa-stained PBLs (d). [file Image1.jpeg]
